# Supplementary material for: Let It Beat: How Lifestyle and Psychosocial Factors Affect the Risk of Sudden Cardiac Death—A 10-Year Follow-Up Study
Source: Int J Environ Res Public Health. 2022 Feb 24;19(5):2627. doi: 10.3390/ijerph19052627 (PMC8909732; doi:10.3390/ijerph19052627)
Supplement: Supplementary file 1 [file ijerph-19-02627-s001.zip › ijerph-1591709-supplementary.pdf]

QUESTIONNAIRE

Instruction: Please write down or circle the most appropriate response.

What is your year of birth? ………………………………………………………….

What is your gender?
Male
Female

Write your height and weight, please……………………cm…………………….…….kg

What is your highest level of education?
Elementary
High school
College/University

What is your occupation?
Employee - please specify……………………………….
Self-employed
Disability pension
Old-age pension
Unemployed - please specify how long …… ……………..
Student
Other - please specify……………………………………..

What is your marital status?
Single
Married
Divorced
Widowed
Other - please specify……………………………..

Please list the diseases you are being treated for:
....
....
....

Please list the medicines you are taking:
………
………
………

Do you have coronary artery disease (a narrowing or blockage of the coronary arteries usually caused by atherosclerosis)? Have you had a heart attack? Have you suffered from angina pectoris?
Yes
No

Has there been a sudden cardiac death or an unexplained death at a young age in your family?
Yes - please specify in whom and at what age ……………………………………………………….
No

Have you had any major life event in the last year (for example death of a loved one, increase in partner quarrels, divorce or separation, violence, major intrafamily conflict, major personal injury or illness, death or major illness of a close family member, a new family member, loss of job or retirement, higher indebtedness, marriage, change residence, extraordinary personal success, etc.)**?**

Yes
No

Do you feel stress at home (feeling irritable, filled with anxiety, or having sleeping difficulties as a result of conditions at home)?

Never
Sometimes
Often
Permanently

Do you feel stress at work (feeling irritable, filled with anxiety, or having sleeping difficulties as a result of conditions at work)?

Never
Sometimes
Often
Permanently

Do you feel financial stress?

Never
Sometimes
Often
Permanently

Do you smoke?

Yes, I do
No, I have never smoked
No, I have stopped - please specify how long..

How often do you sport? (30 minutes to sweat)

Less than once a week
Once or twice a week
At least three times per week

How much of your free time do you spend in front of a TV or computer screen?

Not at all
Less than 1 hour a day
Less than 2 hours a day
More than 2 hours a day

Thank you for completing the questionnaire.
